# Hepatocellular Carcinoma in a Free-Ranging Three-Toed Sloth (*Bradypus variegatus*)

**DOI:** 10.3390/ani12151921

**Published:** 2022-07-28

**Authors:** Alex Junior Souza de Souza, Andreza Pinheiro Malheiros, Victor Lopes da Silva, Tereza Cristina da Silva, Bruno Cogliati, Lilian Rose Marques de Sá

**Affiliations:** 1 Department of Pathology, School of Veterinary Medicine and Animal Science, University of São Paulo, São Paulo 05508-270, SP, Brazil; souzajralex@gmail.com (A.J.S.d.S.); terezacs678@gmail.com (T.C.d.S.); bcogliati@usp.br (B.C.); 2 Hepatology Section, Evandro Chagas Institute, Belém 66093-020, PA, Brazil; andrezamalheiros@iec.gov.br; 3 Prefeitura Municipal de Barueri, Barueri 06401-090, SP, Brazil; victorl_@hotmail.com

**Keywords:** liver, hepatic tumor, neoplasm, xenarthra, pilosa

## Abstract

**Simple Summary:**

Monitoring diseases and lesions in free-ranging and captive wild animals is important for biodiversity conservation and for understanding factors that can impact not only animal health but also human and environmental health. Here, we report for the first time a case of liver cancer in a free-ranging three-toed sloth (*Bradypus variegatus*) and describe the gross, microscopic, and immunohistochemical features of the lesion. The tumor was identified during the necropsy of a three-toed sloth that had to be euthanized due to serious consequences of an accident in the electrical network. Despite not being related to the cause of death of the animal, the type of tumor observed, a hepatocellular carcinoma (HCC), is one of the most frequent hepatic neoplasms in the liver of humans and domestic animals.

**Abstract:**

The increasing interest of tumors in wildlife is important for biodiversity conservation and for monitoring environmental agents and/or contaminants with potential impact on human health. Here we described the occurrence of hepatocellular carcinoma (HCC) in noncirrhotic liver of a free-ranging three-toed sloth (*Bradypus variegatus*) from the Atlantic Forest biome in Brazil. The HCC showed a moderate mononuclear inflammatory infiltrate within the tumor tissue but with no inflammation and fibrosis in the adjacent liver tissue. Upon immunohistochemistry, neoplastic cells were diffusely positive for HepPar-1 and glutamine-synthetase presenting an irregular and random immunostaining pattern; β-catenin was positive in the cytoplasmic membrane of malignant hepatocytes; and cytokeratin 19 immunostaining was restricted to bile duct epithelial cells. The liver tissue was negative for HBV-like and HCV-like viruses assessed by molecular tests. The potential similarity of pathogenesis may reinforce the need for research on environmental and/or infectious agents associated with HCC that may contribute to the understanding of cancer in wildlife.

## 1. Introduction

The Anthropocene is marked by the increasing frequency of cancer in wildlife populations [1] and oncology studies in wildlife have contributed to a better understanding of the multiple mechanisms of cancer development in humans and animals [2]. Furthermore, knowledge about tumors in wild animals and associated factors can be applied as sentinels for environmental contaminants and in the conservation of global biodiversity [2].

In humans, hepatocellular carcinoma (HCC) is the most frequent primary liver cancer [3,4]. Liver fibrosis and cirrhosis induced by chronic liver diseases, hepatitis B virus (HBV) or hepatitis C virus (HCV) infections, alcohol, non-alcoholic steatohepatitis, and aflatoxin B1 are the main factors associated with human HCC [3,4,5].

The incidence, etiology, and risk factors of HCC in animals are not completely understood, but it is postulated that they are similar to humans, considering chronic liver infections, chronic chemical, or heavy metal exposure [6]. Orthohepadnavirus chronic infection in woodchucks [7], chimpanzees [8], and domestic cats [9], as well as hepacivirus infection in chimpanzees [10] and *Helicobacter* spp. in mice [7], are examples of infectious agents associated with HCC in animals. Chronic exposure to aflatoxins in rhesus monkeys [11], copper in dogs [12], hemosiderosis in bats [13], and endocrine-disrupting compounds in fishes’ white suckers [1] are examples of chemical or heavy metals agents associated with HCC in animals.

In animals, the possibility of HCC arising from hepatocellular adenoma is considered, but there is still no consensus on the descriptions and classifications of this tumor progression [7]. The comparative pathology of HCC may contribute to the improvement of criteria for the differential diagnosis of primary tumors in animals and also to the understanding of the risk factors associated with liver neoplasia development [1,7].

Several immunohistochemical and/or molecular markers have been applied for the diagnosis and classification of HCC in humans [3,4,14]. Based on the histopathological, immunohistochemical, molecular, radiological, and clinical profile, at least 12 subtypes of HCC have already been characterized in humans [15]. However, there is not yet a consensus on the use of immunohistochemical and/or molecular panels for the diagnosis and classification of HCC in domestic animals [7,16,17,18].

The present study aimed to describe the first report with anatomopathological and immunohistochemical features of HCC in a three-toed sloth (*Bradypus variegatus*) specimen from the Atlantic Forest biome, Brazil.

## 2. Case Presentation

A free-ranging adult male three-toed sloth, 5.5 kg, was received in emergency medical care, due to an accident in the electrical network that resulted in contracture, paralysis of the thoracic limbs with deep necrosis, palmar ulceration, and severe prostration. The animal was treated with surgical debridement, antibiotics, analgesics, and potent systemic anti-inflammatory and daily curative. Four days later, the sloth presented bilateral extensive tissue necrosis from the hands to the forearms, and euthanasia was performed.

**Figure 1:**
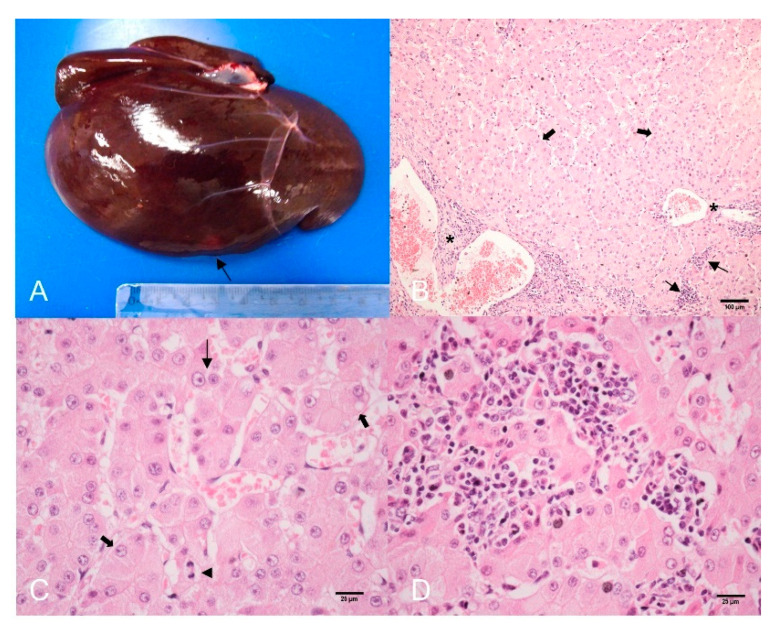
Hepatocellular carcinoma (HCC), liver, three-toed sloth (*Bradypus variegatus*). (**A**) Gross of liver showing HCC as a single whitish nodule (arrow) at the border of the left lobe. (**B**) Trabecular pattern HCC. Note thickened trabeculae containing 4 to >5 cells (thick arrows), lobular inflammatory infiltrate (thin arrows), and in pseudo portal spaces (*). HE. Bar 100 µm. (**C**) Well-differentiated neoplastic hepatocytes showing anisocytosis (thick arrows), anisocariosis (thin arrow), and mitosis figure (arrowhead). HE. Bar 25 µm. (**D**) Lymphocytic inflammatory infiltrate is observed within sinusoids and between neoplastic cells. HE. Bar 25 µm.

At gross, the liver was dark red, enlarged with round edges, and showed a single whitish nodule with poor limits, 1.5 × 1.0 × 2.0 cm, in the ventral caudal region of the diaphragmatic face of the left lobe (Figure 1A). Metastases were absent. Liver fragments were frozen at −70 °C or fixed in 10% buffered formalin and embedded in paraffin, cut in 5 µm sections, and stained with hematoxylin-eosin (HE), Perls, Sirius red, reticulin, rhodamine, and periodic acid Schiff (PAS). Histological evaluation was performed using Eclipse Ni-U light microscope (Nikon, Tokyo, Japan) coupled with a DS-U3 cooled digital camera (Nikon, Tokyo, Japan) and NiS elements F 4.00.00 software (Nikon, Tokyo, Japan) for image capture.

For immunohistochemistry (IHC), 5-μm liver sections were submitted to antigen retrieval for 20 min in a pressure cooker and the blocking of endogenous peroxidase was performed with 6% hydrogen peroxide. Afterwards, the slides were incubated with primary antibodies for the immunostaining of hepatocyte-specific antigen (HepPar-1), β-catenin, glutamine synthetase (GS), and cytokeratin 19 (CK19) overnight at 4 °C (Table 1). The signal was amplified by polymer detection system (Novolink™, Leica Biosystems, Newcastle, UK), visualization was achieved by diaminobenzidine (DAB) chromogen, and slides were counterstained with Harris hematoxylin. Negative controls consisted of omission of the primary antibodies and positive controls were samples of normal tissue (liver, spleen, and lymph node) from dog and another three-toed sloth. Additionally, immunostaining in normal liver tissue adjacent to the HCC lesion was evaluated as an internal reaction control.

A search for Orthohepadnavirus infection was conducted using total DNA purified from frozen liver fragments using the QX DNA High-Resolution Kit (QIAGEN, Hilden, Germany), in a QIAxcel Advanced automated system (QIAGEN, Hilden, Germany), according to the manufacturer’s guidelines. The DNA purified from the sample was evaluated by hemi-nested PCR for HBV-like amplification [19]. Another fragment of the frozen liver was also subjected to total RNA purification using the RNeasy Mini Kit (QIAGEN, Hilden, Germany) and assessed by nested PCR for amplification of hepaci- and pegiviruses (HCV-related virus) [20].

## 3. Results

Microscopically, the neoplastic nodule was non-encapsulated and showed moderate compression of the non-neoplastic adjacent liver tissue (Figure S1). The nodular lesion presented loss of lobular architecture and a trabecular pattern, formed by well-differentiated hepatocytes with frequent binucleations and conspicuous nucleoli, predominantly organized in trabeculae ranging from 2 to >5 cells (Figure 1B) and, less frequently, forming nests. Within the lesion, the portal tracts were absent (only pseudo portal spaces were observed) and few pseudo glands formed by neoplastic hepatocytes, mild ductular hyperplasia, discrete sinusoidal dilatation and fibrosis were randomly observed. There were mild pleomorphism, anisocytosis and anisocariosis, and 9 mitosis figures in 2.37 mm² (corresponding to 10 fields in FN 22/40× objective) (Figure 1C). There was moderate multifocal infiltrate of lymphocytes, few macrophages within sinusoids among neoplastic hepatocytes (Figure 1D) and moderate similar infiltrate in the pseudo portal tracts. Rare neutrophils were seen in the tumor and liver, preserved and interpreted as consequence of sepsis.

**Figure 2:**
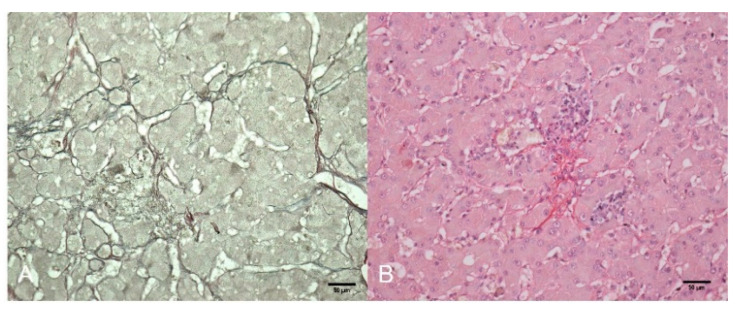
Hepatocellular carcinoma (HCC), liver, three-toed sloth (*Bradypus variegatus*). (**A**) Severe loss of reticulin framework in the thick trabeculae of neoplastic hepatocytes. Reticulin. Bar 50 µm. (**B**) Mild perisinusoidal and periductular fibrosis within the HCC lesion. Sirius red. Bar 50 µm.

Reticulin stain was scanty to absent in several foci within the tumor and marking > 5 cells’ trabeculae (Figure 2A). There was mild perisinusoidal and periductular fibrosis surrounding foci of proliferated hepatocytes by Sirius red stain (Figure 2B) and there was diffuse glycogen deposit in tumor cells. Iron, copper, and lipofuscin were negative in neoplastic hepatocytes.

The edge of the tumor showed a mild deposition of glycogen, lipofuscin, and ferric pigment in compressed non-neoplastic hepatocytes (Figure S1B–D) and non-neoplastic adjacent liver parenchyma (Figure S2A–D) showed an absence of inflammatory infiltrate and/or fibrosis, mild microvesicular steatosis, minimal iron stain, light lipofuscin, mild glycogen, and minimal copper deposition.

Non-neoplastic liver tissue adjacent to HCC showed strong immunostaining for Hep-Par1 in the cytoplasm of hepatocytes; GS was expressed in isolated hepatocytes around the terminal hepatic vein (zone 3); and the immunostaining of β-catenin and CK19 were restricted to bile duct cells (Figure S3). The same pattern of immunostaining was observed in the positive controls (data not shown), certifying the cross immunoreaction of the antibodies with three-toed sloth liver tissue.

**Figure 3:**
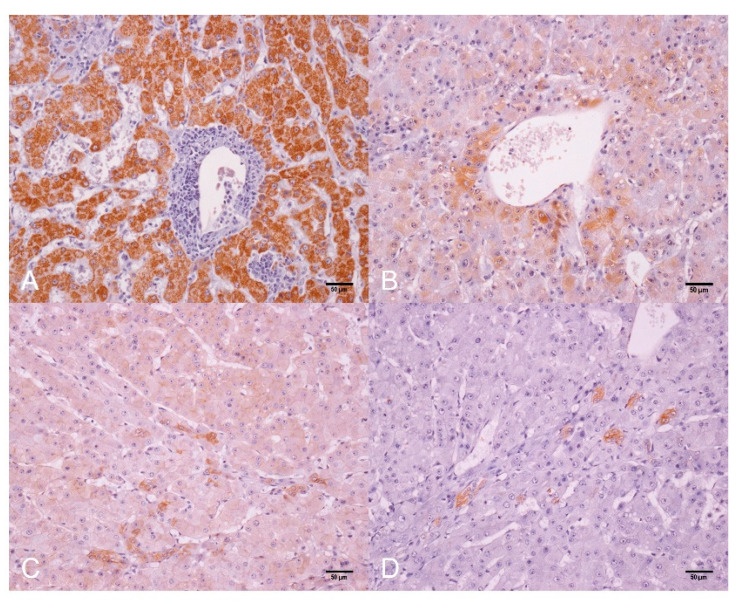
Hepatocellular carcinoma (HCC), liver, three-toed sloth (*Bradypus variegatus*). (**A**) Strong immunostaining of HepPar-1 in the cytoplasm of neoplastic hepatocytes and negative staining in cholangiocytes and inflammatory cells infiltrate. IHC. Bar 50 µm. (**B**) Patchy cytoplasmic immunostaining of GS in neoplastic hepatocytes around the tumor vein. IHC. Bar 50 µm. (**C**) Discrete membranous β-catenin immunostaining in neoplastic hepatocytes and strong positivity in proliferated ducts within HCC lesion. IHC. Bar 50 µm. (**D**) Positive immunostaining for CK19 in biliary duct cells proliferated within the tumor and was negative in neoplastic hepatocytes. IHC. Bar 50 µm. Diaminobenzidine (DAB) chromogen and counterstaining with Harris’ hematoxylin.

Within HCC, the HepPar-1 stain was marked and positively diffused in the cytoplasm of neoplastic hepatocytes (Figure 3A). GS stain was patchy and random in the cytoplasm of hepatocytes (0–10 to >70% of cells) in the tumor (Figure 3B). The β-catenin was mildly expressed in the plasma membrane and cytoplasm of hepatocytes in multiple foci in the HCC (Figure 3C). CK19 immunostaining was positive in the biliary epithelium within HCC (Figure 3D).

Overall, microscopic and immunohistochemical findings characterized the well-differentiated trabecular HCC with lymphocyte infiltrate in a noncirrhotic liver. Samples assessed for HBV-like and HCV-like infection were negative.

## 4. Discussion

HCC was once previously described as an incidental finding in a captive two-toed sloth (*Choloepus didactylus*) specimen from the USA [21], but as far we know, our findings represent the first report of HCC in a free-ranging, three-toed sloth from the Atlantic Forest biome in Brazil.

The left hepatic lobe was affected in the sloth, which is the most frequent lobe affected by HCC in animals [7]. The tumor was composed of well-differentiated cells, with a trabecular pattern and mild fibroplasia as described before in animals [3,4,6,7,22] but differed from HCC in a two-toed sloth, which was larger, showed severe fibrosis, necrosis, hemorrhage foci, and the absence of lymphoid infiltrate.

In animals, the gross morphology of HCC can be nodular, massive, or diffuse; and the histological features of neoplastic hepatocytes in HCC can range from well-differentiated to markedly pleomorphic cells [6,7]. The differential diagnosis between well-differentiated trabecular HCC and hepatocellular adenoma can be challenging and the morphological criteria of trabecular thickness > 5 cells (up to 20) can be decisive for the diagnosis of HCC, since the trabeculae are uniform and have up to 3 cells in hepatocellular adenomas [6,7].

Here, the absence of chronic liver injury and/or repair features adjacent to the nodule ruled out the diagnosis of a regenerative nodule [6,7]. The single nodular HCC was differentiated from nodular hyperplasia and hepatocellular adenoma lesions due to histological malignant features such as absence of portal tracts and loss of lobular architecture; pleomorphism, anisocytosis, anisocariosis, and mitosis figures of hepatocytes; thickened irregular trabeculae with >5 hepatocytes, associated with loss of reticulin framework [3,4,6,7]; and emphasis should be placed on the occurrence of inflammation only within the HCC, which is very uncommon in animals even with HCC in cirrhotic liver and/or chronic hepatitis [6,7,22]. To the best of our knowledge, this is the first report of a HCC in the non-cirrhotic liver of a wildlife animal, associated with the presence of inflammation only within the tumor.

We did not identify any etiological agents that could explain the inflammatory infiltrate within the tumor. In humans, the presence of inflammation only within the tumor is associated with the expression of C-reactive protein and serum amyloid A, which are characteristic of the inflammatory hepatocellular adenoma, a benign subtype that may progress to malignant transformation to HCC in noncirrhotic livers [3,5]. The sloth samples of tumor were negative for both markers (data not shown).

In humans, lymphocyte-rich HCC is a new and rare recognized subtype of HCC, where the main histological feature is the presence of lymphocytes outnumbering tumor cells [3,23,24]. Despite the sloth’s HCC presenting a lower number of intratumoral lymphocytes when compared to human lymphocyte-rich HCC, we cannot exclude the existence of a similar/analogous subtype in animals [3,23,24]. To date, there is still no consensus on risk factors, clinical, anatomopathological, and/or molecular criteria for defining the lymphocyte-rich HCC in humans [3,23,24]. Since the profile of lymphocyte-rich HCC in noncirrhotic livers had not been previously described in domestic or wild animals [6,7,22], we consider that our description will contribute to the comparative pathology of HCC. However, a larger case series of domestic and wild animals needs to be evaluated to determine the existence of lymphocyte-rich HCC subtype in Veterinary Medicine.

HCC in dogs and cats may have variable amounts of ferric pigment [7,22] and the occurrence of hemochromatosis has been associated with HCC among Egyptian fruit bats [13]. The hepatic copper deposit has been associated with chronic hepatitis, cirrhosis, and the occurrence of HCC in dogs [12,22]. Here, negative copper pigment and hemosiderosis within the tumor with minimal deposition in non-neoplastic liver, and the absence of chronic hepatitis and cirrhosis in the adjacent liver ruled out a direct or indirect role of altered iron and/or copper metabolism in the pathogenesis of HCC [6,7].

An HCV-like virus with undetermined biological significance was recently described in three-toed sloths from Costa Rica [25]. However, in the three-toed sloth, the liver was negative for HBV-like and HCV-like viruses. The clinical significance of hepatotropic viruses in the sloth liver still needs further evaluation.

Positive immunostaining for HepPar-1 and a negative stain for CK19 in neoplastic hepatocytes of sloth HCC went as expected and as occurred in HCC of other animals [7,16,17]. Additionally, the immunostaining profile of CK19 in the three-toed sloth HCC was compatible with the profile in well-differentiated/less aggressive HCC in domestic animals [6,7,16,17].

The immunohistochemical profile of patchy GS and membranous β-catenin positive with negative nuclear immunoexpression observed in sloth HCC was similar to the pattern described in well-differentiated HCC in humans with β-catenin mutations [14]. Our study tried to evaluate mutations associated with the expression profile of GS and β-catenin, but it was negative, using standardized protocols for human tissues (data not showed). Based on isolated cases of neoplasms in free-ranging animals, we are aware of the limitations of population inferences of cancer in wildlife. However, we believe that the association of immunohistochemical and genetic markers can contribute to the differential diagnosis of hepatic neoplasms in domestic and wild animals. Indeed, comparative pathology studies in different taxa can also help to understand the hepatocarcinogenesis in wild animals.

## 5. Conclusions

In conclusion, the HCC was the first description of this spontaneous cancer in a free-ranging three-toed sloth, which highlights the need for research to assess the distribution of cancer or benign neoplasms among wild animals in the Atlantic Forest biome as well as to point out environmental and anthropogenic factors related to liver diseases in wild animals as sentinels for human diseases.

## Figures and Tables

**Table 1:** Details of primary antibodies applied for the immunohistochemical evaluation of three-toed sloth hepatocellular carcinoma.

Target Antigen	Clone	Manufacturer	Dilution	Antigen Retrieval Buffer
HepPar-1	OCH1E5	Dako, Carpinteria, CA, USA	1:500	Citrate buffer (pH 6.0)
β-catenin	H102	Santa Cruz Biotechnology, Santa Cruz, CA, USA	1:200	Citrate buffer (pH 6.0)
GS	GS6	Millipore, Bedford, MA, USA	1:500	Citrate buffer (pH 9.0)
CK19	B170	Leica Biosystems, Wetzlar, Germany	1:500	Citrate buffer (pH 9.0)

HepPar-1: hepatocyte-specific antigen; GS: glutamine-synthetase; CK: cytokeratin; GS: glutamine synthetase.

## Data Availability

Not applicable.

## Supplementary Materials

The following supporting information can be downloaded at: https://www.mdpi.com/article/10.3390/ani12151921/s1, Figure S1: Edge of hepatocellular carcinoma (HCC), liver, three-toed sloth (*Bradypus variegatus*).; Figure S2: Non-neoplastic liver adjacent to hepatocellular carcinoma, three-toed sloth (*Bradypus variegatus*).; Figure S3: Immunohistochemistry, non-neoplastic liver adjacent to hepatocellular carcinoma, three-toed sloth (*Bradypus variegatus*).
