# Supplementary material for: Hepatocellular Carcinoma in a Free-Ranging Three-Toed Sloth (Bradypus variegatus)
Source: Animals (Basel). 2022 Jul 28;12(15):1921. doi: 10.3390/ani12151921 (PMC9367539; doi:10.3390/ani12151921)
Supplement: Supplementary file 1 [file animals-12-01921-s001.zip › animals-1732061-supplementary.pdf]

Supplementary Materials

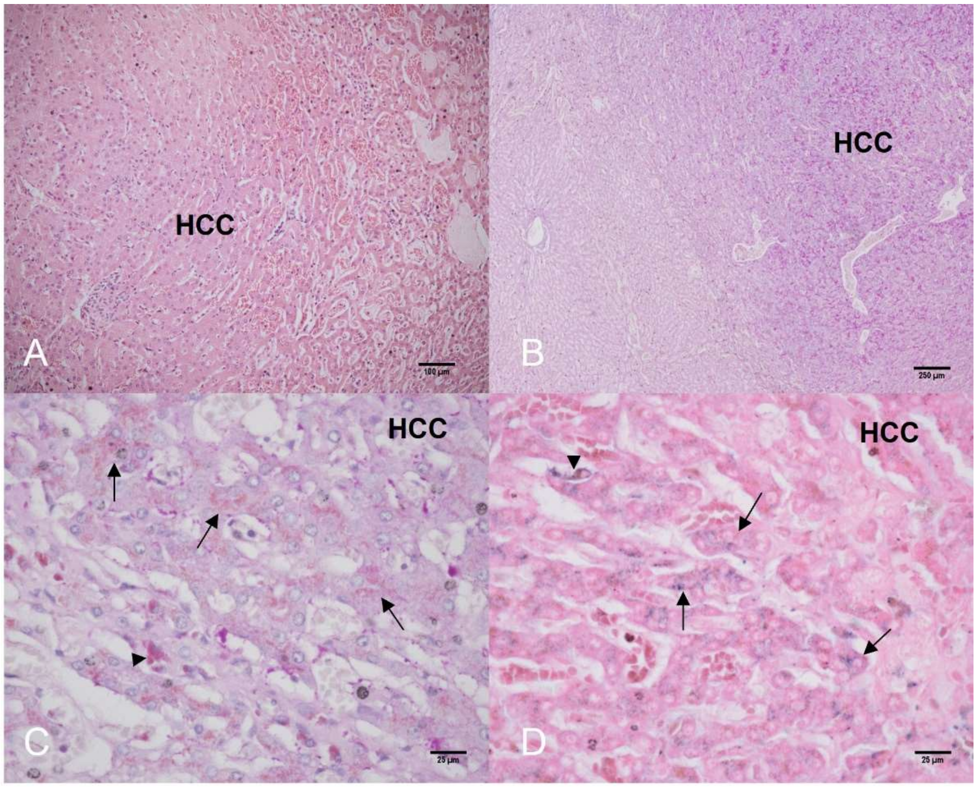

Figure S1. Edge of hepatocellular carcinoma (HCC), liver, three-toed sloth (*Bradypus variegatus*). (**A**) HCC compressing liver tissue on the edge of the lesion. Note dilation and congestion of sinusoids and atrophic hepatocytes. HE. Bar 100 µm. (**B**) Diffuse deposition of glycogen in neoplastic hepatocytes within HCC and mild in adjacent liver parenchyma (lower left corner). PAS. Bar 250 µm. (**C**) Mild lipofuscin (reddish granular pigment) in cytoplasm of Kupffer cells (arrowhead) and non-neoplastic hepatocytes (arrows). PAS. Bar 25 µm. (**D**) Mild ferric pigment (blue granular pigment) in cytoplasm of Kupffer cells (arrowhead) and non-neoplastic hepatocytes (arrows). Perls. Bar 25 µm.

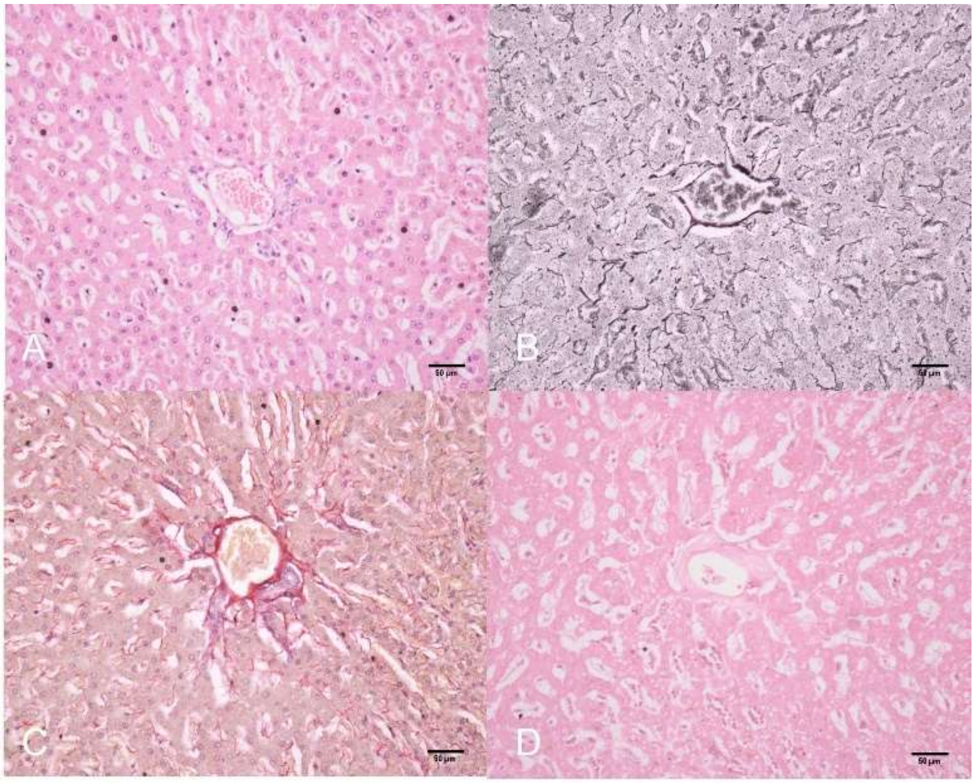

Figure S2. Non-neoplastic liver adjacent to hepatocellular carcinoma, three-toed sloth (*Bradypus variegatus*). (**A**) Regular trabeculae of hepatocytes showing only mild microvesicular steatosis associated to mild sinusoidal dilation. HE. Bar 50 μm. (**B**) Preserved reticulin framework. Reticulin. Bar 50 μm. (**C**) Normal collagen matrix. Sirius red. Bar 50 μm. (**D**) Negative iron deposition in liver tissue. Perls. 50 μm.

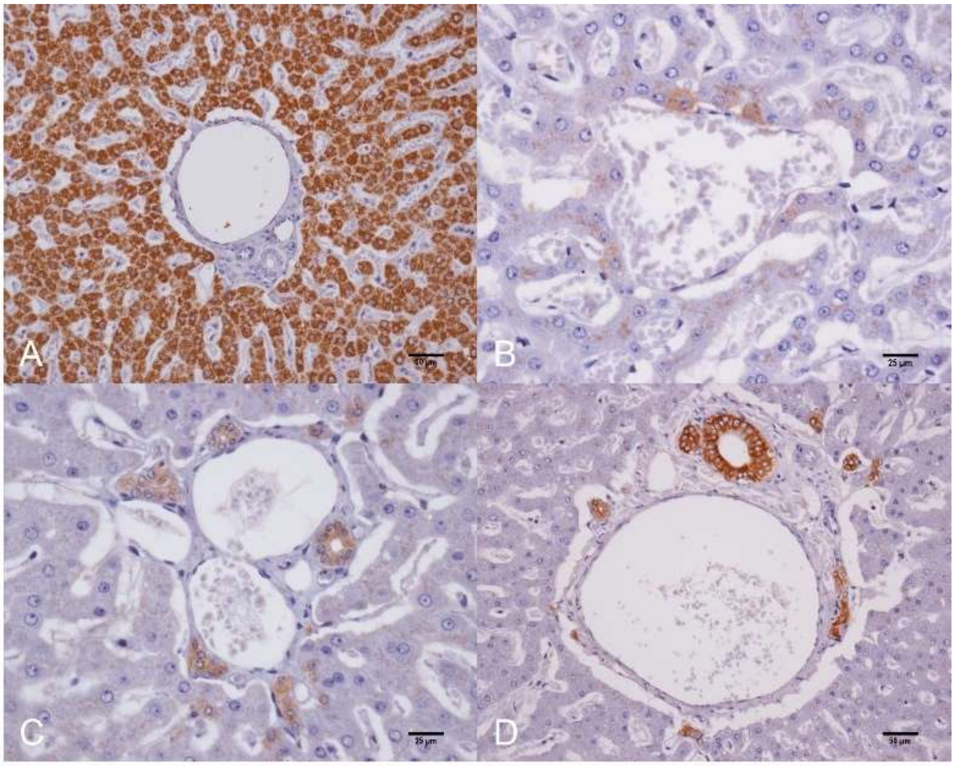

Figure S3. Immunohistochemistry, non-neoplastic liver adjacent to hepatocellular carcinoma, three-toed sloth (*Bradypus variegatus*). (**A**) Strong immunostaining of HepPar1 in the cytoplasm of non-neoplastic hepatocytes. Bar 50 μm. (**B**) Mild immunostaining of GS in isolated hepatocytes around the terminal hepatic vein. Bar 25 μm. (**C**) Mild immunostaining of β-catenin only in bile duct cells. Bar 25 μm. (**D**) Strong immunostaining of CK19 in bile duct cells. Bar 25 μm. Diaminobenzidine (DAB) chromogen and counterstaining with Harris' hematoxylin.
